# Treatise on the Diseases Occasioned by Lead

**Published:** 1840-10

**Authors:** 


### Art. VII.

*Traité des Maladies de Plomb ou Saturnines.* Par L. Tanquerel des Planches, Docteur de la Faculté de Médecine de Paris.—*Paris,* 1839.
*Treatise on the Diseases occasioned by Lead.* By L. Tanquerel des Planches, m.d.—*Paris,* 1839. 2 vols. 8vo, pp. 550, 344.

Although the poisonous effects of the preparations of lead have been known for a long period of time it is only recent researches which have given a definite form and character to the knowledge previously obtained, and enabled us to recognize and to trace these effects with certainty as the result of a specific cause. Perhaps the volumes of M. Tanquerel may be taken as exhibiting, in a more decided manner than has hitherto been done, the invariable relation of certain symptoms known under the name of lead colic and paralysis, with the introduction of this metal in some form or other into the system. Many exceptional cases, in which this peculiar form of colic might be supposed to arise from other causes, are noticed, and upon closer investigation traced to an evident and satisfactory connexion with some preparations of lead, and the whole subject is placed in a clearer light,—the work leaving, in fact, upon most of the leading points, little to be desired.

The treatise of M. Tanquerel comprises separate dissertations upon the following subjects:—1. Lead Colic, ("Colique de Plomb ou Saturnine," Colicum Pictonum, Devonshire Colic, &c.) 2. Rheumatism or Neuralgia from Lead, ("Arthralgie Saturnine.") 3. Paralysis from Lead, ("Paralysie Saturnine.") 4. Loss of sensation from Lead, ("Anæsthesie Saturnine.") 5. Delirium, Coma, &c., from Lead, ("Encephalopathie Saturnine.") These are preceded by a few preliminary remarks upon the effects produced on the system by lead previous to the development of the more decided forms of disease, the induction as it were of a saturnine diathesis, which the author somewhat fancifully terms "*Intoxication saturnine primitive*."

The principal marks by which this state of the system may be recognized are, according to M. Tanquerel, a peculiar bluish or bluish-gray tinge of the gums, which sometimes extends over the mucous membrane of the mouth generally, the teeth at the same time becoming discoloured and affected with caries; a sweetish, styptic, astringent taste in the mouth, with a peculiar fætor of the breath; sallowness of the skin, and dull yellow tinge of the conjunctiva; general emaciation, and small soft compressible pulse and, in some rare cases, a considerable reduction in the number of its beats. Of these symptoms the discoloration of the gums and teeth is the most frequent and the most characteristic. It appears to be owing to the deposition of a very minute film of sulphuret of lead on the mucous surface and on the enamel of the teeth, the former becoming of a bluish slate-gray colour, as before mentioned; the latter of a brown colour, which is deepest at the neck of the tooth or the part in immediate contact with the gum.

This fact of the coloration of the gums by lead has been also noticed by Dr. Schilbach of Neustadt, and by Dr. Burton, of St. Thomas's hospital.* The latter author is inclined to rely on this symptom as an infallible proof of the presence of lead oxide in the system, and thinks that in all cases of illness originating from this oxide, about the symptoms of which some ambiguity exists, an examination of the gums will materially assist in making a correct diagnosis. The importance of attending to the premonitory symptoms generally is shown by a statement made by M. Tanquerel with respect to lead colic, from which it appears that of 1217 cases of this affection coming under his notice, 1195 were previously affected with one or more of the symptoms here mentioned, a timely attention to which, with temporary cessation from work, has in many instances been successful in averting the threatened attack. (p. 190.)

I. Lead Colic. The definition given by M. Tanquerel of this affection is as follows: "A neuralgia of the digestive and urinary organs produced by the introduction and absorption into the system of lead in the molecular state. This neuralgia is characterized by severe and continuous pains of the abdomen, becoming more acute by paroxysms or crises, diminished or but little if at all augmented by pressure, and accompanied by rigidity and retraction of the abdominal parietes, by obstinate constipation, vomiting or nausea, eructations of intestinal gases, loss of appetite, dysuria, slowness and hardness of the pulse, and by agitation and anxiety. Thus," he adds, "there is in the lead colic exaltation of the

* Froriep's Notizen for October, 1839. London Medical Gazette for January, 1840.

sensibility with perversion of the contractility and of the secretions of the diseased organs." (p. 22.)

It would not be difficult to show that this definition or rather brief summary of the characters of lead colic, is as liable to objection as those of certain preceding authors. To this, however, we should not have thought it necessary to refer had it not been for the very questionable hypothesis of the nature of lead colic put forward in it. Neither the character of the pain, the symptoms existing simultaneously with it, the subsequent degeneration into paralysis,* nor the methods of treatment most commonly found effective in its cure, afford any countenance to the idea that the disease is a neuralgia. In the historical sketch which follows we have a condensed account of the progressive steps made in the recognition of the disease, from the first faint traces to be discovered in the writings of the older physicians down to more modern times, in which an assemblage of symptoms similar to those now known to arise from the introduction of lead into the system was first clearly described. Nicander, whose writings next to those of Hippocrates are perhaps the oldest extant, gives a sufficiently exact though concise description of the symptoms of lead colic, and recognizes the metal both as the cause of these and of the paralysis in which they not unfrequently terminate; but with this single exception, the agency of the metallic poison in the production of the peculiar effects from time to time observed seems to have been lost sight of. Accordingly, even in later times, though the disease itself was recognized, we find it described under various names, some of which point out an endemic origin, (colica Pictonum, colique de Normandie, Devonshire colic, &c.;) others connect it with certain trades (painter's colic, hutten katze, colique des fondeurs, &c.;) others refer to causes of a different description or of less definite character, (colique vegetale, colique minerale.)

The work of M. Merat (Traité de la Colique Metallique) is not free from this last objection, and it is due to M. Tanquerel to state that he has done much towards clearing up and simplifying the true causation of this and other diseases arising from lead, by the investigation of cases apparently exceptional, and the excluding of other agents whether mineral or vegetable from any share in the production of like phenomena.

"Lead, then, and its compounds must actually penetrate into our economy in order to give rise to the lead colic, that is, to an affection consisting in a special modification of organs more or less remote from the part to which these poisons can be primarily applied. These substances after having been previously deposited on some part of the human body, are taken up by the capillaries and carried into the circulation, by which means they arrive at the abdominal organs and disturb their functions......It is only as a consequence of their absorption that the particles of lead can give rise to colic, constituting an essential difference between this disease and the inflammatory poisoning produced by the ingestion of a large quantity of the preparations of lead into the stomach. In this latter case the poison is not absorbed, but irritates the membrane with which it is in immediate contact, and may inflame and ultimately destroy it." (pp. 49, 50.)

M. Tanquerel mentions among the preparations of lead which have

* It should be observed, however, that M. Tanquerel would scarcely admit that paralysis is in any case a consequence of the colic.

been known to give rise to the lead colic : 1. Metallic lead. 2. Lead in combination with oxygen ; the sub-oxide, massicot, litharge, and minium or red lead. 3. Combinations of lead with various acids; borate of lead, sub-carbonate, ceruse or white lead, phosphate, chromate, and nitrate of lead, the acetate or sugar of lead, and the sub-acetate or extract of lead, and Goulard's extract. 4. Sulphuret, chloride, cyanide, and silicate of lead. And 5. The alloys of lead with tin (solder), antimony, copper, silver, and gold. It is, however, questionable whether metallic lead ever really gives rise to colic, or other diseases produced by the action of this poison on the human body, as there is reason to conclude from the rapid action of the oxygen of the atmosphere and other physical agents upon this metal, that previously to its absorption it may become converted into an oxide or a carbonate, sulphuret, or some other compound.

The question of the mode by which the poison is introduced into the animal system is one of the highest importance, since upon its correct determination depends in a great measure the efficacy of the preventive measures employed to counteract the noxious effects of the several processes of manipulation to which the workmen in leadworks of various descriptions are exposed. Without going so far as to say, with M. Tanquerel, that absorption by the skin never takes place, or at least is never the means by which the poison of lead is introduced into the system in sufficient quantity to give rise to colic, it may, we think, be admitted that, compared with other modes of access, the cutaneous absorption of lead is of extremely infrequent occurrence.

In support of his opinion upon this point M. Tanquerel cites with justice the frequent and long-continued application of plasters and lotions containing lead in surgical practice. We cannot, however, concede to him that his three experiments on two dogs and a rabbit at all tend to confirm his views. In one of the dogs a drachm of carbonate of lead in combination with axunge was rubbed into the skin daily for a period of eight days; in the second dog lead plasters were applied to the abdomen and chest previously deprived of the hair ; and in the rabbit an ointment composed of an ounce of litharge and an equal quantity of axunge was rubbed into the inner part of the thigh, which had also been previously shaven, the frictions in this last experiment being performed three times a-day until the ointment was expended, which occurred in twelve days. Both the skin of the first dog and that of the rabbit were blackened by the solution of sulphuret of potash, but no effect was observed to be produced on the health of the animals subjected to experiment.

"In the face of like facts, some positive, others negative," says the author, "the choice, it appears to me, is easy. What confidence can be placed in the simple assertions put forth by the writers of past ages to prove that lead in contact with the cuticle is capable of giving rise to lead colic, when we bring them together with a great number of negative facts, observed with much care by enlightened men living at a period when everything referring to observation is carried on with an exactness and closeness of attention of which a great number of our predecessors seldom thought." (p. 59.)

Were the negative facts referring to the absorption of lead by the skin of no higher value than M. Tanquerel's positive facts, as detailed in the very superficial and trivial experiments referred to, they would indeed possess very little weight, and the most determined opponent of the doctrine of cutaneous absorption would hesitate before he drew any inference from three experiments extending over a period of from eight to twelve days, in the face of a long series of cases in which persons have been exposed for months to the reception of this poison in every possible way, and others in which it has been actually administered internally in considerable doses continued daily for a much longer period without the production of colic or any other injurious effect.

Contenting ourselves, however, with enforcing the necessity of attending in all cases to the frequent cleansing of the hands and skin generally by those engaged in the manipulation of lead, we proceed to the consideration of what we believe, with M. Tanquerel, to be by far the most frequent if not the sole method by which the poison of lead becomes introduced into the system—absorption by the mucous membranes in the processes of ingestion and inhalation.

The author adduces many striking facts illustrative of the danger of employing preparations of lead as remedies without due caution; among others, he quotes a singular case from M. Alphonse Devergie in which a single grain of the acetate produced colic, increased by a second dose, and rendered so violent by a repetition of the same quantity a third time as to give rise to a judicial investigation. The preparer of this medicine was accused of negligence in making up the pills, but on chemical examination they were found to contain only one grain each. On the other hand, M. Fouquier gave the same preparation in the dose of eighteen grains daily for several months; and M. Barbier, of Amiens, exhibited as much as 240 grains in thirty-seven days without even the slightest symptom of colic being produced. It is remarkable that in the face of these cases and others to the like effect, M. Tanquerel should for one moment have placed any confidence in the experiments before mentioned. Without, however, throwing doubt upon the occasional administration of lead in large doses and for a considerable length of time without injurious consequences, he expresses himself as being persuaded that preparations of lead taken internally as medicines give rise to colic more frequently than is generally believed. It is sufficiently evident, however, that the personal experience of M. Tanquerel respecting the medicinal qualities of lead is most defective. "I have only seen," he says, "these substances administered four or five times, and twice I have noticed the appearance of colic and other lead affections." (p. 70.) It is absurd to found any conclusions on such a bald experience as this; but we must allow that they are borne out by the testimony of the late Dr. Andrew Duncan, whose opportunities of observation were much more extensive. We have ourselves had frequent occasion to employ the acetate of lead in cases of internal hemorrhage, sometimes in frequently repeated doses and continued for some time, and have never seen any injurious effects arise from its use; but we would nevertheless impress the caution of watching, in all instances where lead is given internally, for the slightest manifestation of its peculiar effects, and the necessity of immediate suspension of the remedy when such occur.

The lead colic and other diseases resulting from the absorption of lead into the system are more frequently induced by the ingestion of various alimentary matters contaminated, either by accident or design, with this deleterious metal. An excellent account of the accidental contamination of water from leaden cisterns, pipes, &c., as well as of the adulteration of cider, perry, wines, and other fermented liquors, will be found in Dr. Christison's Treatise on Poisons, with which work, as well as with the writings of Sir George Baker, Dr. Wall, Dr. Percival, and other English authorities, we are glad to observe M. Tanquerel shows a competent acquaintance. Much important matter from these sources is quoted or referred to, which as being already well known to the English reader, may here be passed over. There can be no question but that in many instances colic is induced among workmen employed in leadworks and trades in which lead is used from the want of due care in the eating of their food, particles of lead being thus frequently conveyed into the stomach with the aliment; but the inhalation and consequent absorption by the pulmonary mucous membrane of the fine particles diffused through the atmosphere in the state of impalpable powder, in many of the operations carried on in the several departments of the works, would seem to be the most frequent cause of lead colic. The liability to colic as a consequence of the inhalation of lead is not, however, confined to those actually engaged in the manipulation of this metal; and the author relates instances of this affection induced by sleeping in apartments recently painted. Among these is the case of Dr. Corsin, of La Villette, near Paris, who after having occupied for two nights a chamber newly painted with white lead, was attacked with all the symptoms of lead colic. After some days of suffering in the abdomen, the head became affected, epileptic fits, alternating with delirium and coma, supervened, and rapidly proceeded to a fatal termination. (p. 86.)

The following is a list of the occupations of 1213 persons affected with lead colic, and is stated to be drawn up from actual observation of the cases during a period of eight or nine years ("from 1831 to 1839,") all, with the exception of nine, having been received at the hospital of La Charité. To this list we have added, for the convenience of comparison, those of the persons suffering from other forms of lead disease, subsequently given by the author in different portions of his work.

| | Colic. | Other Diseases. | | | | Total. |
|---|---|---|---|---|---|---|
| Manufacturers of white lead | 406 | 220 | 31 | 9 | 25 | 691 |
| Ditto red lead | 63 | 104 | 6 | 3 | 5 | 181 |
| Ditto massicot | 12 | 7 | .. | .. | .. | 19 |
| House painters | 305 | 168 | 22 | 6 | 20 | 521 |
| Coach painters | 47 | 33 | 4 | .. | .. | 84 |
| Ornamental painters | 33 | 25 | 5 | .. | .. | 63 |
| Porcelain painters | 3 | .. | .. | .. | .. | 3 |
| Gilders | 1 | .. | .. | .. | .. | 1 |
| Painters or varnishers on metal | 2 | .. | .. | .. | .. | 2 |
| Coloured paper makers | 2 | .. | .. | .. | .. | 2 |
| Colour grinders | 68 | 43 | 6 | .. | 3 | 120 |
| German and glazed cardmakers | 19 | 7 | 1 | 1 | 3 | 31 |
| Sword-belt makers | 2 | .. | .. | .. | .. | 2 |
| Perfumers | 2 | .. | .. | .. | .. | 2 |
| Earthenware potters | 54 | 33 | 5 | .. | 2 | 94 |
| Dutch-ware potters | 7 | .. | .. | .. | .. | 7 |
| Refiners | 25 | 14 | 3 | .. | 2 | 44 |
| Plumbers | 14 | 10 | 3 | 1 | 3 | 31 |
| Tinmen (etameurs) | 8 | 3 | .. | .. | .. | 11 |

| | Colic. | Other Diseases. | | | | Total. |
|---|---|---|---|---|---|---|
| Puttymakers | 4 | .. | .. | .. | .. | 4 |
| Block-tin workers (ferblantiers) | 4 | .. | 1 | .. | .. | 5 |
| Jewellers, goldsmiths, trinket-makers | 4 | .. | .. | .. | .. | 4 |
| Copper-founders | 2 | 1 | .. | .. | .. | 3 |
| Bronze-founders | 1 | .. | .. | .. | .. | 1 |
| Type-founders | 52 | 38 | 4 | .. | 2 | 96 |
| Printers | 12 | 8 | 3 | .. | 1 | 24 |
| Small-shot manufacturers | 11 | 6 | .. | 1 | 2 | 20 |
| Lapidaries | 35 | 27 | 3 | 2 | 3 | 70 |
| Cutters and polishers of crystals | 3 | .. | 1 | .. | .. | 4 |
| Plate-glass workers | 2 | .. | .. | .. | .. | 2 |
| Manufacturers of acetate, nitrate, and chromate of lead | 10 | 5 | 4 | .. | .. | 19 |
| | 1213 | 752 | 101 | 23 | 72 | 2161 |

In addition to the occupations enumerated in the preceding table, M. Tanquerel mentions, as liable to suffer from the action of lead, workmen employed in lead mines; manufacturers of litharge, of glass, and of porcelain; glaziers, &c.; but of cases of colic or other disease arising from lead, amongst workmen of these descriptions, he has had no personal experience. It will be observed that this list differs considerably from those obtained from the records of the hospital of La Charité by M. Merat, in being much more extensive and complete, and embracing a far greater number of instances of the occurrence of lead-disease. Yet the author states that all the workmen whom he has seen attacked with lead colic had been in some way or other in contact with lead, though, according to M. Merat, persons employed in various copper-works, and even in other trades and occupations in which no lead is used, are also liable to be attacked. Among these are mentioned seven button-makers, five brass-founders, four braziers, and one copper-turner, in all seventeen belonging to trades exposed to the influence of copper; and four varnishers, two gilders, two locksmiths, and twelve others of various occupations, all of whom, however, though not actually engaged in leadworks, might possibly in other ways have been exposed to the influence of lead. Another and more important source of error in the statistical statements referring to this subject is pointed out by M. Tanquerel, who shows that without other sources of information little dependence can be placed upon the registers of La Charité. "Thus, for example," he observes, "all the workmen employed in the manufactories of white lead and minium only work there accidentally, being unable from a temporary scarcity of work to carry on their habitual occupation. It results, therefore, that when individuals of this description, the most numerous class of the sick, present themselves at La Charité attacked with lead colic, they are almost always entered in the registers of the establishment under their orginal trade of mason, terrace-maker, plasterer, shoemaker, founder, distiller, marine, soldier, &c." (p. 94.)

It is scarcely necessary to follow the author through all the details of the several professions and occupations which he enumerates, and upon each of which he affords much valuable information. The general result is that all those parts of the various leadworks and manufactories in which lead is employed, where the particles of metal are diffused through the atmosphere in the state of impalpable powder, are particularly noxious and that on the contrary, where the lead is worked in its solid and massive state, or prevented from escaping in the form of dust or powder by the use of water in such parts of the several processes as admit of it, injurious effects are far less frequently experienced. This fact is especially shown in the white-lead manufactories. In those of Clichy and Courbevoie, in the vicinity of Paris, from which the greater number of cases are sent to the hospital of La Charité, two methods of manufacturing the ceruse are followed, in each of which, during certain parts of the operation, a constant dissemination of the carbonate of lead in finely-divided particles through the atmosphere of the apartments takes place. The average period which the workmen continue at their work before experiencing an attack of colic is, at Clichy, fifty-one days, at Courbevoie, fifty-seven. M. Tanquerel assigns as the chief reason of this difference between the two manufactories, the greater extent of the former; more workmen being employed and a much larger quantity of white lead being made at Clichy than at Courbevoie, and the atmosphere being in consequence more loaded with the metallic particles. He is also inclined to attribute it in part to the carrying on at Clichy of the several processes of the manufacture in one large apartment, in consequence of which all the workmen become exposed to the same highly-charged atmosphere; whereas at Courbevoie a system of seclusion to a certain extent is adopted, and those only are exposed to the inhalation of the white-lead dust who are employed in the various operations of crushing, picking, grinding, sifting, packing, &c. It must, however, be remarked, that there is also a trifling difference in the processes themselves, by which at the manufactory of Courbevoie one of the most noxious of the earlier operations is dispensed with. In several of our own manufactories various improvements have recently taken place, by which much of the danger is obviated; the practice of dry grinding, one of the most injurious from the vast quantity of dust generated and diffused by it, is now abolished, and the process performed under water. In a large manufactory at Portobello, the rolling or crushing of the plates of lead incrusted with the carbonate formed upon them in the first process of the operation, by which the white lead is separated, is performed under water or with damping, and the only operations now considered dangerous at the Portobello works are the emptying of the drying stove, and the packing of the white lead in the barrels, and even in these the dust is kept down as much as possible by the floor being maintained constantly damp. "By these precautions," says Dr. Christison, "and by care being taken to make the workmen wash their hands and faces before leaving the works for their meals, and to administer a brisk dose of castor oil on the first appearance of any complaint of the stomach or bowels, the manufacturer succeeded in extirpating the colica pictonum entirely for several years. A few years ago it appeared again to a limited extent among the work-people, apparently in consequence of the rules as to cleanliness not having been so carefully enforced." (Christison on Poisons. 2d ed., p. 506.)

The beneficial effects of an appropriate system of seclusion are also observed in the manufacture of red lead; accordingly at Clichy, where leadworks of this description are extensively carried on, and where the several parts of the process are followed in two apartments only, the workmen being kept constantly exposed to an atmosphere charged with the emanations or the dust arising from the packing, powdering, calcining, drying, and reverberating operations, the proportional number of sick is much more considerable than at the manufactories of MM. Tourrasse and Guizet, in which each operation is carried on in a separate apartment and a smaller quantity of the red lead is produced. (p. 119.) The more dangerous parts of the operation are those enumerated above, and in the order mentioned; but here, as in the white-leadworks, improvements have been effected in this country by altering the construction of the furnaces,—an additional chimney being placed in front of the drawing aperture, through which a strong current of air carries off the finer particles raked out from the furnace, which under the older plan were diffused through the apartment. (Christison, op. et loc. cit.) The average term at which the workmen become affected with lead disease in the Parisian red-leadworks is forty-five days; so that according to this method of calculation, the manufacture of red lead would seem to be even more deleterious than that of ceruse.

The most numerous class affected with lead colic next to the white-lead manufacturers is that of the house-painters; it must not, however, be inferred on this account that any relation in point of actual danger exists between these occupations. The number of persons employed in the preparation of white lead is not more than 106, while no less than 406 cases of colic in individuals of this class are entered in the table. As this table extends over eight or nine years, the annual average of cases is between forty-five and fifty, or about one half of those engaged in the manufacture. The number of house-painters in Paris, according to an estimate of M. Tanquerel, obtained from his enquiries among the the masters, foremen, workmen, police-registers, &c., amounts to 5000 or 6000, who in the favorable seasons, that is, in spring, summer, and autumn, are in constant employ. The annual average of cases of this trade entered in the table is between thirty-five and forty, or about one in 150 of those employed. M. Tanquerel states that of those who exercise this profession during thirty or forty years, nearly a fourth become attacked at least once in the period. Some can scarcely continue even a few days at the work before being affected with colic; others do not experience an attack under twenty or thirty years, while the greater number are fortunate enough to escape altogether. (p. 124.) The more injurious parts of the trade are stated to be those in which particles of lead are diffused through the atmosphere, as the painting with spirits of turpentine, or dead white, as it is technically termed in this country, the preparing of the colours, and the dry scraping and rasping of walls, previously painted, with pummice-stone, &c. Carriage-painters, of which there are about 1000 employed in Paris, are relatively less subject to colic than house-painters, which is attributed to their never carrying on their trade in confined apartments, to the quantity of colour used being less, to the materials employed being prepared with varnish, &c.

One of the most pernicious, perhaps it might be said the most pernicious, among the occupations in the foregoing table, is the making of German and glazed cards. Of twenty-four persons employed in the manufacture of the "*cartes d'Allemagne*," not more than a fifth escape being attacked with colic. The materials employed to colour the paper are preparations of lead, ceruse being used for the white, and red lead or litharge being added when red tints are required. A kind of paste is prepared by mixing together white lead, water, and some sort of glue

(*colle*), with the occasional addition of litharge, red lead, or other colouring matters. To apply this coating to the pasteboard it is boiled, and the workmen are constantly surrounded with an atmosphere of vapour in which particles of white lead are suspended; in addition to this, every second or third day they scrape and clean their work-benches covered with the dried ceruse, thus diffusing this material through the apartment in the state of impalpable powder or dust. It is to be hoped that the use of the glazed cards, the preparation of which is so highly injurious to those engaged in it, will in future be dispensed with, the more especially also since accidents have arisen from young children having sucked the poison, where they have been incautiously permitted to obtain possession of them.

M. Tanquerel states that if plumbers, or those employed in the melting and working of lead in its metallic state, are careful not to touch their food with unwashed hands, they are never attacked with colic; but he gives a remarkable instance of several boys employed at the royal manufactory in the Rue des Prouvaires in unrolling a considerable number of rolls of lead in a small room, becoming suddenly affected. It seems that they were engaged the whole day in this operation in the midst of a cloud of dust filling the air of the room in which they worked. On the morrow with one exception only, they were all attacked with lead colic. (p. 150.) Type-founders would seem to suffer much, since of 100 workmen engaged in type-founding, one fourth had been attacked with colic once or oftener; this is, however, attributed to want of care in the management of their food, which many of them are accustomed to prepare and to eat in their workshops. Some compositors also who have contracted the injurious practice of putting the types in the mouth while correcting their pages, become liable to the disease. Copper-founders, however, notwithstanding what has been advanced by Merat and others, are stated by M. Tanquerel not to be liable to colic; and he asserts that the instances of colic supposed by these authors to arise from the action of copper had not been studied with sufficient care. Had the different processes in the copper-works where the workmen became attacked with colic been closely investigated, it would have been found, he thinks, as he has himself observed in like cases, that the only operations in which the workers in copper contract the colic are those which require the employment of a small quantity of lead. (p. 154.)

It is unnecessary to pursue these remarks further; we turn, therefore, to the consideration of the subject of predisposition, with the view of attempting some estimate of the extraneous causes which may have a tendency to favour or impede the development of the symptoms of lead-poisoning in the system. The cases of lead colic and arthralgia occurring at different seasons were as follows:

| | Colic. | Arthralgia. | | Colic. | Arthralgia. |
|---|---|---|---|---|---|
| January . | 67 | 32 | July . | 190 | 93 |
| February . | 77 | 45 | August . | 127 | 89 |
| March . | 95 | 62 | September | 92 | 63 |
| April . . | 99 | 69 | October . | 84 | 54 |
| May . . | 115 | 74 | November | 78 | 45 |
| June . . | 137 | 91 | December | 59 | 38 |
| | | | | 1217 | 755 |

A trifling difference will be observed here from the general numbers given in the table of occupations, but not sufficient to affect in any way the result of a progressive increase of the respective diseases from January to July, and a progressive diminution of the same from July to the close of the year. A similar result, although the cases are not given in monthly returns, was noticed in the paralytic affections; thus of the 102 instances reported, twenty-eight occurred in spring, thirty-six in summer, twenty-six in autumn, and only twelve in winter. This increased frequency of diseases from lead-poisoning during the summer months is attributed by M. Tanquerel partly to the greater activity of the works at that time,—the white-lead manufactories, for example, being sometimes stopped for months at a time in the winter, and more than one half of the house-painters being out of employ during the same season. Notwithstanding the operation of these circumstances, however, the author states it as the result of his enquiries that, *cæteris paribus*, the number of workmen sick is always greater during the hot than during the cold seasons of the year, and concludes, therefore, that heat acts as a predisposing cause in the generating of lead disease, either by favouring the dissemination of the lead itself, or more especially by rendering the system more permeable to the introduction of the poison.

The following table shows the ages of those affected:

| Years. | Colic. | Arthr. | Paral. | Anæsth. | Enceph. |
|---|---|---|---|---|---|
| From 5 to 10 | 8 | 2 | .. | .. | .. |
| 10 „ 20 | 80 | 52 | 2* | 1 | 4* |
| 20 „ 30 | 244 | 167 | 24 | 3 | 20 |
| 30 „ 40 | 445 | 290 | 36 | 10 | 30 |
| 40 „ 50 | 277 | 163 | 28 | 6 | 12 |
| 50 „ 60 | 118 | 63 | 8 | 2† | 5 |
| 60 „ 70 | 39 | 18 | 4 | .. | 1 |
| 70 „ 80 | 6 | .. | .. | .. | .. |
| | 1217 | 755 | 202 | 22 | 72 |

But little information as to the effect of age in predisposing to the reception of the poison of lead or to the development of lead disease can, however, be drawn from a statement of this imperfect character, other details being required for comparison before any judgment could be attained: such are the ages of the workmen generally, the relative period of exposure, the intensity of that exposure, and many other conditions respecting which we have no information. It may be observed, however, that five or six children, employed in a manufactory of fancy papers and cards belonging to M. Hangrand, are stated to be frequently attacked with colic, while the adults in the same manufactory are more rarely so; on the other hand, old persons have been met with by the author who have not been attacked with colic after attaining an advanced age, though still under the same circumstances of exposure as when formerly the subjects of disease. (p. 180.)

We find little more to remark upon this subject, except that here, as in other diseases of totally different character, habits of intemperance, and

* Under 20. † Between 50 and 70.

even occasional excesses powerfully predispose to the reception of the morbid influence and the development of its effects.

"Generally, sober persons are spared by the disease, or if attacked it is much later, and the attacks are renewed only after pretty long intervals. ........ In a good many cases we have remarked that the disease showed itself on the morrow and even on the second day after a debauch. Monday is the day on which the working people of Paris most frequently commit excesses in drinking, and accordingly we have many times seen that a greater number of sick are received into the wards of La Charité on the Tuesday and Wednesday." (p. 155.)

The lead colic is usually marked by severe twisting or griping pains; retraction and hardness of the abdominal parietes; a more or less constipated state of the bowels; nausea and vomiting, with eructations of wind and occasionally hiccough; loss of appetite, thirst, and diminution or suppression of the secretion of urine. Some of these symptoms, however, are of far more frequent occurrence than others, and may therefore be considered as more decidedly characteristic of the action of lead. M. Tanquerel thinks that the pain is not only the most important symptom, without which, indeed, the disease cannot exist, but that it is also the first in point of appearance. Usually, he observes, it is situated at the umbilicus, less frequently at the epigastrium or hypogastrium, and more rarely still is it found to occupy the region of the kidneys, the hypochonders and lateral parts of the abdomen, or the chest. The median line of the abdomen was observed to be the usual seat of the pain, and even in the comparatively rare instances, when the whole abdomen became affected, the parts most pained were the umbilicus, epigastrium, and hypogastrium. (p. 195.) In no case was the colic-pain observed to follow the precise course of the colon. The intensity and duration of the pain are of every degree, from the most trivial and transient up to paroxysms of long continuance and of such extreme severity as to deprive the wretched sufferer of all consciousness of what is passing around him. Pressure commonly relieves it, and it is a frequent practice among those attacked to have recourse to heavy weights placed upon the abdomen, with the view of obtaining some respite from the severity of their sufferings. This is not, however, universally the case, since of the patients observed by the author 300 experienced no alleviation of the pain by pressure, while in 175 the pain was slightly, and in thirty-nine considerably, increased under compression. Thus in almost two thirds of the whole number of cases, the pain was diminished by pressure; in about a fourth it was neither diminished nor augmented; and in the sixth part nearly there was an increase. (p. 202.) Constipation is of almost constant occurrence, the bowels having been confined in 1140 out of the 1217 instances. In thirty-three of the remainder the bowels were regular, in twenty-five there was diarrhœa during the first two days of the attack, and in nineteen throughout the whole course of the disease. (p. 203.) The evacuations were generally deficient for several days, and in one case the constipation had lasted for a fortnight. That the relaxed state of the bowels in those who were affected with diarrhœa did not depend upon any complication was shown, as the author thinks, by the effect of the usual purgative treatment, under which it completely and quickly disappeared. Six cases of colic are subsequently referred to, as having occurred during the prevalence of a slight epidemic diarrhœa, in which the epidemic constitution was observed to exert its influence in so far modifying the usual symptoms of the disease, but in these also the purgative treatment proved equally efficacious in the removal of the diarrhœa as in those before alluded to.

It was thought by Merat that the retraction and hardness of the abdomen were in proportion to the severity of the pain; this, however, is questioned by M. Tanquerel, who expressly states, as the result of the observations collected by himself, " that a constant relation of proportional intensity between these two data cannot be established." (p. 205.) In a succeeding paragraph, however, we find him, in accordance with the views advanced by M. Merat, saying: " In general the more acute the colic pain, the more marked are the hardness and contraction of the belly. Some exceptions to this rule, of which it is difficult to detect the cause, exist." (p. 208.) We conclude that neither M. Merat nor M. Tanquerel, in laying down a general proposition, even in sciences more exact than that of medicine, would ever think of asserting that exceptional cases or residual phenomena may not be found which prevent us in the present state of our knowledge from making the rule absolute. The rule of M. Merat may, therefore, be substantially true, although, from the interference of causes not accurately defined, exceptional cases, the rationale of which we are unable to appreciate or explain, may occur. To determine how far the muscular coat of the bowels participates in the rigidity which affects the abdominal muscles, the author examined the rectum of a great number of persons suffering under attacks of lead colic, and concludes as the result of his trials that the anus and rectum are always more or less affected with a spasmodic constriction analogous to cramp.

" If at the immediate commencement of a paroxysm in violent lead colic, the forefinger is attempted to be introduced into the rectum, a considerable difficulty is at first experienced to its entering the bowel, arising from the sphincter, which is so strongly contracted as to occasion a sense of tightening and numbness to the finger, as if it were held in a vice. By pushing somewhat strongly the resistance from the anus is at length overcome, and the finger enters the lower part of the rectum, the walls of which are found approximated. For some lines and even an inch beyond, the opposite walls of the bowel are still observed to be almost in contact, and if it is wished to penetrate further, they must be separated mechanically by the finger. If the finger is retained in this manner during several paroxysms of the colic, the same sensation of tightening and numbing is very sensibly felt at the instant of access throughout the whole extent of the rectum in contact with the finger, as at the circumference of the anus, but in a less degree. During the remission, this sensation of constriction always continues, though much less marked, to return again with increased strength at the paroxysm." (p. 210.)

M. Tanquerel has observed the occurrence of nausea and vomiting in a very large proportion of the cases, the former being much more frequent than the latter, and generally preceding its occurrence. Only twenty-two cases where the epigastrium was affected without being attended with vomiting were noticed, and in these the pain was slight. The matters vomited were of a leek-green colour, of viscid consistence, of very fetid peculiar smell, and of an extremely bitter metallic taste, which was by some compared to that of lead, by others to verdegris, according to the notion entertained as to the cause producing this attack. (p. 213.)

It should be stated that M. Merat was inclined to attribute the frequency of vomiting to the peculiar treatment adopted, with which opinion, however, M. Tanquerel does not coincide. Eructations were very common; the passage of flatus by the anus rare. Hiccough was observed in 115 of the cases, which were for the most part of great severity. The tongue was usually moist, red at the edges and tip, slightly coated, white and sometimes yellow at the middle and at the base; pretty frequently it was observed to be somewhat swollen, ("*plus grosse que dans l'état normal,*" p. 215.) Some of the patients complained of dryness of the mouth; others on the contrary said that the saliva was increased in quantity; but not one instance of salivation or ptyalism was observed by the author without complication with stomatitis, angina, &c. There was generally considerable thirst, the indulgence of which not unfrequently afforded some relief to the pain; in other cases, however, the swallowing of any liquid produced a sense of suffocation. The appetite was very rarely preserved. Very frequently a considerable degree of irritation of the urinary organs accompanied the paroxysms of colic; the desire to pass water was urgent; but although repeated efforts were made by the patient for this purpose, there was no excretion of urine during the paroxysm, or it passed only drop by drop. During the remission of the pain the urine flowed more freely, and sometimes after the trial had been repeatedly made for a length of time ineffectually, it would all at once pass in considerable quantity. (p. 219.) The cause of this symptom is not very apparent; possibly various causes may in different cases concur in giving rise to it. Stoll and Dance attribute it to spasm of the urethra, but M. Tanquerel observes that he has never seen distension of the bladder produced by the accumulation of a large quantity of urine in these cases as happens in paralysis of that organ. He conjectures that the suppressed evacuation of urine may depend upon defective secretion, which will especially be the case when the colic is seated in the region of the kidneys. It is not improbable that both these causes are in operation, together with spasm of the ureters and of the bladder itself, by which, at the same time that the propulsion of the urine into the bladder is impeded, the desire to pass what may find its way during the remission of the spasm into that organ becomes urgent. Accordingly, as we have recently had occasion to observe, a sense of distension, fulness, and weight arises in the region of the kidneys and along the whole course of the ureters, which is in some cases particularly distressing, but yields readily as soon as a copious flow of urine has taken place.

Jaundice has been noticed by the author in fifty-one cases supervening in the midst of "horrible suffering without the slightest alleviation of the symptoms. Thirty-five times it coincided with pains seated in the right hypochonder and fifteen times with pains in the epigaster." Care must be taken not to confound attacks of jaundice of this nature with the habitual discoloration of the skin, &c., or jaundiced state induced by the long-continued and gradual action of lead on the system.

Notwithstanding the severity of the pain, the circulation is rarely accelerated, and in many instances preserves its natural characters. Of the 1217 cases reported by the author, the pulse was in 678 beween thirty and sixty, in 376 from sixty-five to seventy, and in 125 from eighty to 100; in the remaining thirty-eight cases there was a complication of the disease with inflammatory symptoms, which accounted for an accelerated rate of the pulse observed in these instances. (p. 228.)

A marked character of lead colic is the striking alteration of the expression of the countenance, which indicates extreme anxiety and the most intense suffering. "It is almost impossible," says M. Tanquerel, "to mistake this expression when it has been once observed, although difficult to convey an idea of it in words. The eyes are deeply sunk in the orbits, or sometimes thrust prominently forwards from their cavities; they are surrounded with a bluish circle, muddy, wild, and with the other parts of the face in constant motion, vividly expressive of the different degrees of pain experienced by the patient. The nose is somewhat pinched; the cheeks become hollow; and in short the muscles and features of the face generally are in a state of tension and retraction analogous to what occurs in those of the abdomen." (pp. 232-3.) The voice sometimes becomes husky and whispering, the respiration more or less oppressed, the general strength annihilated, while the intellectual faculties and *morâle* are entirely thrown off their balance.

M. Tanquerel attempts to establish certain varieties of lead colic depending upon the seat of the pain, its intensity, &c. He thus distinguishes the umbilical, epigastric, hypogastric, and renal forms; the mild, moderate, and violent; the acute and the chronic. These distinctions are, however, trivial, and for the most part of little practical importance, with the exception, perhaps, of the last. He also points out certain complications with other diseases which are of frequent occurrence, more especially those with the affections arising from lead described in the sequel, and with diseases of the intestinal canal and of the urinary organs in general. It appears that in 525 of the colic cases arthralgia existed at the same time; in forty-four cases there was paralysis, and in thirty-five encephalopathia simultaneously present. It is worthy of remark that the number of cases of lead colic admitted at La Charité during the year in which the cholera appeared at Paris was excessively small, and that the manufactories of white lead and minium of Paris and its vicinity had fewer workmen attacked with the lead colic than in preceding years: six of the patients were attacked with cholera. (p. 248.)

The progress and duration of lead colic present considerable variety; three stages are distinguished, those of invasion, increase, and decline. Without entering at any length into the separate consideration of these several stages it may be briefly observed that the invasion is frequently sudden, though generally preceded by the premonitory symptoms before noticed, and sometimes by slight or wandering pains in some part of the abdomen, and according to some authorities by constipation. The stage of increase, or rather of persistence, is characterized by constipation, paroxysms of pain, with remissions, &c. of varying severity, but with little or no change in the actual state of the disease until its transition into the third stage or that of decline, which is almost always rapid, the disease ceasing almost entirely in the course of a single day or even a few hours.

"The constipation gives way, or commonly has given way, the evacuations taking their usual course; the abdomen regains its softness and becomes convex; the sphincters relax; the nausea and vomiting subside; the flatus begins to take its usual course downwards; the appetite becomes good; the fetid taste and breath disappear; the urine flows easily and in abundance; the pulse recovers its frequency, its regularity, and its accustomed softness; a restorative sleep dissipates the fatigue, the sense of soreness or general uneasiness resulting from the violent shock recently inflicted on the whole system. Finally, this same day, or at least the following, the remains of the colic completely disappear and the patient is cured." (p. 251.)

Sometimes, however, in the midst or even at the close of the stage of decline, the symptoms reappear in all their severity; a relapse, which is commonly attributable to error in diet, to a renewed exposure to the emanations arising from lead, or some other imprudence on the part of the patient. When persons who have contracted the disease are enabled, in consequence of its mildness to continue at their work, and of course are still exposed to the source from which it originally sprung, the duration of the symptoms, unless controlled by appropriate treatment, seems indefinite. M. Tanquerel has witnessed instances of this description where the disease has lasted for whole years. Where the work is laid aside and the affection left to the efforts of nature, it may continue indefinitely for twelve or fifteen days, for months, for years; in a few instances it disappears spontaneously, in from two to eight days, being generally of longer duration when severe than when moderate or slight. When subjected to active treatment by the employment of drastic purgatives repeated daily, the average duration, according to the author, is not more than three or four days, computing from the actual commencement of these remedial measures. (pp. 254-5.)

The diagnosis of lead colic from other affections, more especially from diseases resulting from other metallic poisons, from the so-called vegetable colic, from gastro-enteralgia, inflammation of the abdominal viscera, organic lesions of the abdomen, ileus, from colic arising from the presence of worms, concretions, collections of hardened fæces, &c., is carefully drawn. Of these the inflammatory forms of disease will be distinguished by the presence of fever, the quickened circulation, the intolerance of pressure, &c.; the organic lesions, concretions, &c., by close examination of the abdomen, and by the seat of the pain. This last is often either confined to one particular spot, the immediate site of the lesion, or of the obstruction whatever may be its nature, or follows the course of some part of the intestinal canal, whereas the pain in lead colic is usually seated in the median line. It is, however, only by a careful consideration of all the symptoms and a close comparison of them with those of lead colic in all its forms, that some of the more obscure cases can be accurately made out. The colic from copper and its compounds, according to M. Tanquerel, differs from that of lead, in never extending beyond the bounds of the abdomen, at the same time that it is generally diffused over the whole of this part, in the pain being always increased by pressure, in the abdomen being somewhat softer and less retracted, in the absence of nausea and vomiting, which are very rarely observed in colic from copper, &c. The pain arising from copper also never gives rise to that peculiar state of agitation and constant change of position and expression which is so characteristic of lead-colic. Instead of the bowels being constipated, as is usually the case in the latter disease, there is a relaxed state of them with abundant glairy, greenish, frequent evacuations, sometimes accompanied by tenesmus; the secretion and excretion of urine are little affected, and the fetid breath, sweet taste, and slate-coloured tint of the gums, together with the sallowness of complexion and emaciation are absent. The colics of Poitou and Devonshire have been sufficiently elucidated, and ascertained to arise from the use of lead; the colic of Madrid, the symptoms of which present great analogy with those of lead colic, is attributed by Luzuriaga and Hernandez to this poison; the French physicians residing in Spain are, however, mostly of opinion that it is owing to improper and acescent vegetable diet and exposure to evening exhalations. M. Tanquerel is disposed to agree with his countrymen, and endeavours, unsatisfactorily as it appears to us, to make out a distinction between the two affections. The subject requires further investigation by unbiassed and competent observers, who should have opportunities not only of noting the symptoms accurately and fully, but of investigating the previous circumstances of the persons suffering under attacks of the disease. From the statements of M. Alfaro it would seem that several different affections, such as enteritis, colitis, dysentery, nephritis, hepatitis, and other diseases of the abdominal viscera, are confounded under the term colic, by the people of Madrid, and that the descriptions of Luzuriaga and Hernandez refer not to all affections commonly classed as colic of Madrid, but to the lead colic, as it is frequently observed in this capital amongst house-painters and other workmen engaged in occupations connected with the use of lead. The colic of Normandy also requires further investigation, as the statements of M. Vasse, a physician at Rouen, scarcely warrant the inferences drawn from them by M. Tanquerel. Previously to quitting the subject of diagnosis, we may observe, that there is an excellent tabular view of the distinguishing characteristics of lead colic and gastro-enteralgia arranged in parallel columns, which is, however, too long for quotation.

The termination of lead colic, according to M. Tanquerel, is, notwithstanding the exaggerated statement of Tronchin, rarely fatal. It must not, however, be inferred from this that the prognosis is favorable in an inverse ratio; for though the attack may not end directly in the death of the patient, as a consequence of the severity of the abdominal symptoms, a fatal result may, and often does ensue from the simultaneous progress or the subsequent development of paralytic or cerebral disease. Of the author's cases ten proved fatal, of which number six were affected with cerebral symptoms, simultaneously with those of colic, two died from paralysis of the intercostal muscles, one from pneumonia, and in one only was death produced by the abdominal affection alone. In this case the patient had been labouring for three months under colic-pains of extreme severity, and notwithstanding the employment of every mode of treatment, at length sunk under the continued suffering, the victim "of a species of painful consumption," (p. 299.) Twenty fatal cases were observed by Dubois and Burette, at La Charité, in a period of thirteen years, and sixty-four fatal cases by Gardanne, during twelve years, but the cause of death is expressly stated by Dubois and Gardanne to have been convulsions, epilepsy, lethargy, apoplexy, &c. In three fatal cases, mentioned by De Haen, and in two mentioned by Combalusier, there were epileptic convulsions and delirium. Doasan lost one after several attacks of epilepsy. M. Merat reports five deaths, not one of which arose from the abdominal affection; and in five cases, observed by M. Andral, there was either delirium, convulsions, coma, or asphyxia. The total number of cases of colic, observed by M. Tanquerel and the authors above mentioned, is 4809, of which 111, or about one in forty-three terminated fatally; but, with one exception only, the fatal result is said to have been owing either to the cerebral affection, to paralysis of the respiratory muscles, or to some accidental complication with diseases, with the production of which the poison had no concern, (pp. 300-1.) It is from the presence of these complications, then, or from the tendency to the cerebral or paralytic forms of lead-disease, that an unfavorable prognosis is to be drawn; and it should be borne in mind that in instances which are purely and simply cases of colic, the whole system is under the influence of the deleterious agent, and consequently that the head or the respiratory muscles may become affected either in the progress or even in the decline of the abdominal symptoms.

Little or no light has been thrown upon the pathology of lead colic by microscopic investigations. The author has collected together forty-nine cases of lead colic, uncomplicated with other diseases, ("colique saturnine non compliquée,"—not complicated, we presume, with diseases not arising from the action of lead,) in which the appearances have been noted down with sufficient precision. In twenty of these no alteration was found in the intestinal canal, or only such traces of congestion as are met with in the greater number of subjects submitted to examination after death, and in which, during life, no functional lesion of the digestive organs had been ascertained. In five cases partial softening, without other alteration, was met with in the most dependent parts of the intestines, a purely cadaveric change, which has been observed in a multitude of instances. Six times there was partial or general thickening of the digestive tube, a lesion which, according to M. Tanquerel, is also met with indiscriminately in pathological examinations, ("dans toute espèce d'autopsie.") Hypertrophy of the glands of Brunner was observed in seven cases, and in three of these there was also enlargement of those of Peyer. In sixteen instances a drawing together or thinness, (un tassement ou un rétrait) of the intestinal convolution was observed, that is, a contracted or constricted state of its coats, as Merat, Leroux, and De Haen describe it, but which M. Tanquerel, influenced by his views as to the nature of the disease shortly to be adverted to, is very unwilling to call it. He considers, that to attribute this state of the intestine to contraction of its parietes is altogether hypothetical, because "slight traction or insufflation readily makes the diminution of caliber in the intestine to disappear. (p. 318.) We are not disposed to contest the point with M. Tanquerel that this contracted state of the intestine is actually the cause of lead colic, but we may observe that admitting, as he does, that it forms one of the anatomical characters of the disease, and that it is found in a third part of the post-mortem examinations, considering also that the leading symptoms of lead colic admit of occasional remissions, and that the fatal result is almost invariably attributable to affections of other organs of the body, that consequently the anatomical characters cannot always be expected to remain at the moment of death if of a nature which is not necessarily permanent, it is at least as consistent with the facts observed, as the gratuitous assumption of the disease being a neuralgia of the grand sympathetic, to which not one pathological fact lends the least countenance. It is true, that upon one single occasion, an increased development of the ganglia of the great sympathetic nerve was ascertained by the author to exist, but he himself admits that this change should be considered as an effect of the symptoms observed during life, and not as their anatomical cause. The kidneys and bladder were, with the exception of some vascular congestion, commonly found nearly of their natural appearance, though in one of M. Tanquerel's own cases, in which there was considerable constriction, or "*tassement*," as he terms it, of the folds of intestine, the bladder also seemed contracted, (*retirée sur elle-même*), and was of very small size.

It has been attempted to detect the presence of lead in the excretions, fluids, and tissues of those affected with lead colic; but, with the exception of a recent analysis by M. Alphonse Devergie, without success. The subject of experiment was a colour-grinder, who had followed that occupation for eighteen months; he was affected with colic, arthralgia, and delirium, having had three previous attacks of colic during the eighteen months. The parts submitted to analysis were the stomach, intestines, and fecal matters, the gall-bladder containing some bile; the kidneys, the bladder, the lungs, weighing twenty-two ounces three drachms; a portion of the brain and of the muscular substance, seven ounces of the blood, and about two grains of the black matter collected from around the neck of the teeth. All these organs afforded traces both of lead and copper, the latter being in very small proportion, but the lead being seven or eight times more abundant than M. Devergie had discovered in persons dying from diseases unconnected with lead poisoning. The process followed was that of incineration. After having dried the animal matter it is reduced to charcoal, calcined in a porcelain crucible, and washed repeatedly. The incinerated matter is then treated by hydrochloric acid; a part of the acid is evaporated, water is added, and this aqueous fluid treated by sulphureted hydrogen; a chocolate brown or black precipitate is formed, according as the copper or the lead predominates. This is collected in a porcelain vessel and treated by nitric acid; the mixture is diluted with water, filtered and evaporated over a gentle fire; the product contains the two metals in the state of a salt. The lead may be procured in the metallic state, by subjecting the sulphuret to the action of the blow-pipe. (tom. i. p. 325.; t. ii. pp. 401-6.) It is scarcely necessary to remind our readers that Dr. Mibmer and Professor Christison have succeeded in detecting lead in some of the tissues of animals poisoned by that metal; the former in the liver, the muscles, and especially in the spinal cord; the latter in the lumbar and dorsal muscles. Their experiments, however, do not exactly fulfil the conditions requisite to make them comparable with colic and other affections arising from the absorption of lead into the human body, and are open, as Dr. Christison himself suggests, to the source of fallacy arising from the now ascertained presence of traces both of copper and lead in the animal solids and fluids generally.

The section devoted to the investigation of the seat and nature of the disease need not occupy us long, the views advocated by the author being entirely hypothetical, and attempted to be supported by distorted or erroneous statements. He sets out by endeavouring to show that the colic is seated in those organs to which the great sympathetic nerve is distributed, and then draws a parallel between the symptoms of this affection and those of neuralgia of various parts, from which he infers that lead colic is a neuralgia of the great sympathetic nerve. "In the diseases termed neuralgia," he observes, "pain is the characteristic symptom; it is acute, subject to exacerbations, and even to intermissions; far from being augmented, it is alleviated by pressure, and follows ordinarily the course of some of the great nervous trunks." (p. 329.) Those who have been accustomed to see much of neuralgia facialis, and other forms of this distressing class of affections, will scarcely be prepared to believe either that the pain is alleviated by pressure, or confined to the great nervous trunks. Often, indeed, when the patient is quiescent, a paroxysm will be induced by the slightest touch, and the course of the smaller nerves is frequently marked out and described with the greatest accuracy from the shooting of the pain along them, by persons suffering under neuralgia, who are entirely unacquainted with their distribution. It is also asked, whether the muscles of the pained parts in neuralgia of the face and of the lower extremities do not contract, do not apparently occupy a smaller space, especially during the exacerbations of pain? (p. 332.) Certainly nothing but the most determined adherence to preconceived opinion could so far have obscured the reasoning and comparing faculties of M. Tanquerel, as to lead him to the supposition that any analogy exists between the strongly-marked spasmodic contractions of the abdominal muscles in colic, and the state of the muscular system in parts affected by neuralgia. But it is foreign to our object here to examine into the merits of these or other hypothetical views of the nature of the disease advanced by De Haen, Astruc, Laennec, Barbier of Amiens, Dubois, Merat, and many other authorities; perhaps the opinion advocated by Ilsmann, Hoffman, and others, that lead colic consists in a spasmodic contraction of the bowels, notwithstanding the assertion of M. Tanquerel that the constriction of the intestine is a consequence and not the cause of the pain, is as near the truth as any which have hitherto been advanced.

The treatment recommended by the author is that followed at La Charité with its modifications, but especially the administration of croton oil, repeated so as to produce its full cathartic effect; he gives, however, some curious details of the administration of other remedies, which are worthy of some consideration. Of thirty-one cases, in which nothing but the ordinary tisane of the French hospitals was given for twelve days, three were cured by the fourth day, two between the fifth and eighth days, ten between the eighth and twelfth days, and one on the thirteenth day; the remaining cases were subjected after the twelfth day to the purgative treatment, under which they were quickly cured. M. Gendrin having stated that he had succeeded in curing more than 300 cases with the sulphuric acid administered in the form of lemonade, ("*la limonade sulfurique*,") a statement, the accuracy of which M. Tanquerel impugns in no very measured terms, fifty-three cases of colic were, at the instance of M. Tanquerel, submitted by MM. Andral, Dalmas, and Sandras, at the hospital La Charité, to this treatment. After a miserable statement of the prolonged sufferings of some of these unfortunate individuals, sixteen of whom laboured under colic in its most severe form, two becoming affected with convulsions, and nine, after from twelve to fifteen days of treatment getting worse, we find the physicians to whose care they were intrusted, to ease their consciences, ("*pour l'acquit de leur conscience*,") at length laying aside the *imonade sulfurique*, and having recourse to purgatives, under which the cure was effected in from one to five days from their commencement. M. Tanquerel coolly remarks, "We have not been able to experiment with this medicine in a greater number of instances from fear of the development of cerebral or spinal symptoms, and also because it is painful to see the unfortunate persons suffering for a long time when they might be cured in a few days under other treatment." (p. 355.) Happily such practices as these are unknown in the public establishments of our own country; but we should fail in our duty were we not to express our utmost disgust at this wretched trifling with the lives and sufferings of our fellow-creatures, under the pretence of alleviating the maladies to which they are often, from dire necessity, exposed. Similar atrocities were perpetrated in trials with nux vomica, but we abstain from presenting the revolting statement to our readers.

The peculiar treatment of this disease, for which the La Charité has been so famous, has been established upwards of 200 years, and is still followed with certain modifications, by the present physicians, and with distinguished success. It consists mainly of purgatives, with occasional emetics, anodynes, and sudorifics. The following is the method, as prescribed at present.

1*st day.** Aqua cassiæ cum granis (1); simple sudorific tisan (2);

* The following are the exact formulæ as given by M. Tanquerel:

| | K. | Gr. | Decig. |
|---|---|---|---|
| 1. *Eau de casse avec les grains.* | | | |
| ℞. Décoct. de tamarin (64 gr.) ............ | 1 | | |
| Emétique (Ant. Tart.) .................... | | | 15 (3 gr.) |
| 2. *Tisane sudorifique simple.* | | | |
| ℞. Décoction de gayac ordinaire ......... | 1 | | |
| 3. *Lavement purgatif des peintres.* | | | |
| ℞. Infusion de séné ............................ | | 500 | |
| Sulfate de soude ........................ | | 16 | |
| Electuaire diaphœnix .................. | | 32 | |
| Jalap pulverisé ............................ | | 1 | 3 (24 gr.) |
| 4. *Lavement anodin.* | | | |
| ℞. Huile de noix ............................ | | 125 (℥iv.) | |
| Vin rouge .................................. | | 314 (℥x.) | |
| 5. *Bol thériacal.* | | | |
| ℞. Thériaque ................................ | | | 4 (ʒj) |
| Extrait d'opium ........................... | | | 0,05 (1 gr.) |
| 6. *Eau bénite.* | | | |
| ℞. Eau commune ............................ | | 500 | |
| Emétique (Ant. Tart.) ................... | | | 25 (5 gr.) |
| 7. *Tisane sudorifique laxative.* | | | |
| ℞. Infusion de séné et décoction de gayac | 1 | | |
| 8. *Potion purgative.* | | | |
| ℞. Infusion de séné ........................... | | 125 | |
| Electuaire diaphœnix } Sirop de nerprun ... } āā ........ | | 32 | |
| Jalap en poudre ............................ | | 1 | 3 (24 gr.) |

purgative injection (3) in the morning; anodyne injection (4) in the evening; and three hours after a bolus of theriac and opium (5).

*2d day.* Aqua benedicta (6); simple sudorific tisan; purgative injection; anodyne injection; theriac and opium.

*3d day.* Sudorific laxative tisan (7), two glasses; simple sudorific tisan; purgative injection; anodyne injection; theriac and opium.

*4th day.* Purgative draught (8) in the morning; simple sudorific tisan; theriac and opium.

*5th day.* Sudorific laxative tisan, two glasses; simple sudorific tisan; purgative injection; anodyne injection; theriac and opium.

*6th day.* Purgative draught in the morning; simple sudorific tisan; theriac and opium.

*7th day.* Sudorific laxative tisan; simple sudorific tisan; purgative injection; anodyne injection; theriac and opium.

Sometimes the emetic is repeated several times, and the dose of the antimony is doubled or even tripled.

During the first two or three days a very low diet (diète sévère) is enjoined; broths (bouillons) are allowed on the fourth or fifth day; and afterwards the diet is gradually improved. (pp. 384-6.)

This practice, although strongly savouring of the continent, does not essentially differ from that mostly followed in this country, namely, a combination or alternation of cathartics with opium, hyoscyamus, and other anodynes. For our own part, we have ever experienced in the cases which we have had occasion to see, the most satisfactory effects from this mode of treatment, aided by warm baths, warm anodyne fomentations, the occasional application of leeches, and the employment of all those means which are fitted to soothe and give comfort to the mind as well as to the disordered state of the nervous and muscular systems of the sufferer.

II. Lead Rheumatism or Neuralgia. The term *arthralgia*, employed by the author to designate this affection, which he defines as signifying "neuralgic pains in the limbs from lead" is an objectionable one, as, from its previous use in medical language, it conveys the meaning of the affection being one of the joints; the sense, however, in which it is employed in the work under notice, is to designate "acute pains in the limbs, unattended by redness or swelling, not following the exact course of the nerves, continuous, but becoming more acute in paroxysms, diminished by pressure, augmented by motion, and accompanied with disturbance of the motive functions, such as cramps, hardness, and tension of the pained parts." (p. 493.) It is in short nothing more than the spasms or cramp-pains of the lead colic affecting the muscles of the limbs instead of those of the abdomen, and is most commonly a mere extension of that disease, although occasionally observed separate. It has not been generally recognized as a distinct disease by authors, although we occasionally find it described or alluded to, and not unfrequently, as a rheumatic affection. According to M. Tanquerel, arthralgia is with the exception of colic the most frequent result of lead poisoning; he states that he has had occasion to see 755 cases, (752 in the table of occupations, see p. 331), of which number 201 were uncomplicated; in the remaining 544 it was associated either with colic, with paralysis, or with cerebral disease. As a general observation it may be stated that the liability of workers in lead to contract this affection is in direct proportion to their liability to suffer from colic; there is, however, one remarkable exception, the reason of which is not very obvious, in the case of the manufacturers of red lead. By reference to the table of occupations which we have before given, it will be noticed that the red-lead-works afforded sixty-three cases of colic, or about one twentieth of the whole number; whereas the number of cases of arthralgia from the same manufactories was 104, or nearly one seventh. It should be observed also, that sixty-eight of the 201 uncomplicated cases of arthralgia, that is, one third of the number, occurred in red-lead manufacturers.

The pain, which is the most important symptom, occupies most frequently the lower extremities, then the upper extremities, the loins, the parietes of the chest, the back, and the head. Of the 755 cases, in 485 the pain was confined to the lower extremities, in eighty-eight to the upper extremities, in eighteen to the loins, in five to the chest, in four to the back and neck, and in three to the head. In 108 it was simultaneously experienced both in the upper and lower extremities, in thirty-five in the trunk also, and in nine the head as well as the limbs and body generally was affected. (p. 503.) The flexor muscles are, according to M. Tanquerel, more frequently the seat of pain than the extensors, the affected muscles in either case being strongly and spasmodically contracted, and their powers of motion greatly impeded; an exacerbation of the pain often occurs during the night, but there is neither preternatural redness, heat, or swelling of the parts, and the circulation is stated to be for the most part undisturbed. Yet we are also told that in fifty-five of the cases of simple arthralgia, that is, upwards of one fourth of the number of cases of this description, the pulse was found to be hard, slow, vibrating, and in seventeen of them irregular. (p. 510.)

The arthralgia from lead is distinguished from acute rheumatism by the absence of any swelling or redness of the joints, by the pain being relieved rather than increased on pressure, by its sudden and complete intermissions, and by the want of fever and of complication with pericarditis and endocarditis; we cannot, however, coincide in the opinion of the author, that the diagnosis is further established by rheumatism usually attacking one member only or the arm and leg of opposite sides, the arthralgia almost always affecting two parallel members. Neither are we disposed to think that chronic rheumatism is so essentially erratic, changing from one limb to another, as to enable us to distinguish between this affection and the arthralgia from lead, whose locomotive inclinations are exerted, we are told, only within the ring fence of the limb in which it has taken up its abode. The effects of pressure and the stiffness of the joints in the former of these diseases are much better points whereupon to found a diagnosis. From simple neuralgia the course of the pain, which in arthralgia rarely follows that of the nerves, is sufficient to distinguish it, even in the opinion of M. Tanquerel, who nevertheless elsewhere asserts that these pains as well as those of the abdominal affection are neuralgic in their nature; and from syphilitic pains in the bones it is sufficiently distinguished by these last following the course of the bones, and being usually attended by thickening of the periosteum or the characteristic nodes.

No appreciable change was observed, either in the spinal marrow or in the local seat of pain, in any of the fatal cases in which arthralgia had been present. In the instance before alluded to, as having been the subject of experiment by M. Devergie, the muscles of the calf of the leg were analyzed, and the presence of lead in these parts was detected. With respect to the nature and seat of this affection, M. Tanquerel thinks that the disease is a neuralgia of the filaments of the nerves of sensation, differing only from lead colic in the part which it occupies. The same treatment has been recommended in arthralgia as in the colic from lead, but, according to this author, is not followed by equal success. He advises the employment of sulphureous baths daily for seven or eight days, from five to six ounces of the sulphuret of potash being used in the preparation of each bath. Of eighty-six individuals thus treated eighty were cured in from four to five days, whereas of eighty persons treated by purgatives and opiates, fifty-eight only were cured in from six to eight days, the remaining twenty-two, having experienced no relief, got rapidly well under the use of the sulphureous baths. (p. 523.)

III. Lead Paralysis. Under this term M. Tanquerel describes the loss or diminution of voluntary motion with its accompanying symptoms, &c. arising in persons subjected to the action of lead, reserving for separate consideration the loss or diminution of sensation from the same cause under the term anæsthesia. Paralysis has been recognized as an effect of lead upon the human system from the earliest times, although, as in the case of lead colic, it is only recently that definite knowledge has been attained respecting its true characters, causation, and general history. It is, however, to the author of the work before us that we owe the most precise details upon the subject. He considers this affection under the several heads of the history, causes, premonitory symptoms, symptoms, and varieties, diagnosis, progress, terminations, and prognosis, anatomical characters, seat and nature, and lastly treatment.

The absorption of lead or its compounds into the system is stated to be the sole efficient cause of this description of paralysis, and it is very properly observed that what is termed mercurial paralysis is altogether a different affection from paralysis, although by some authors classed with it. M. Tanquerel remarks that of upwards of fifty cases of mercurial tremors observed at the hospital of La Charité, in no one instance was paralysis seen to supervene, and his researches among the workmen generally employed in the manipulation of this metal, as well as of copper, tin, arsenic, and other metallic substances, lead to the conclusion that lead is the only mineral capable of producing paralysis properly so called. The same conditions which influence the production of lead colic exert also a similar influence in the production of lead paralysis; and although there are cases on record which would seem to favour the idea of a paralytic effect being induced by the action of lead on the skin, yet it must be admitted that the more usual mode in which it exerts its deleterious agency is by its gradual introduction through the digestive and respiratory passages. Generally it requires a considerable time before the symptoms of paralysis show themselves, those only becoming affected who have been long engaged in leadworks, and who have already suffered from repeated attacks of colic; exceptional cases, however, are found, as the following table of the periods at which the 102 cases of paralysis seen by the author occurred, sufficiently shows, (vol ii., p. 19):

| Duration of work. | No. of Sick. |
|---|---|
| 8 days . . . | 3 |
| From 15 days to 2 months . | 11 |
| „ 1 year to 5 years . | 36 |
| „ 5 years „ 10 „ . | 20 |
| „ 10 „ „ 15 „ . | 12 |
| „ 15 „ „ 20 „ . | 12 |
| „ 20 „ „ 25 „ . | 7 |
| 52 „ . | 1 |
| | 102 |

Huxham, De Haen, Stoll, Andral, and others mention the occurrence of paralysis from lead without any previous attack of colic; M. Trousseau is stated to have observed two cases of paralysis of the wrists at the hospital of Tours in persons who had never been affected with colic, and we have ourselves had occasion to observe two similar instances, in one of which, we may remark, the action of the lead would seem to have been purely local. Of M. Tanquerel's cases, also, fourteen had not suffered from colic before the coming on of paralysis; twenty-five had experienced one attack of colic; fifteen had suffered from two attacks; nine had had colic three times previously to the occurrence of paralysis; eight four times; and seven five times; eighteen had experienced as many as from six to ten attacks; four from twelve to twenty; and one person became the subject of paralysis after having suffered no less than thirty times from colic. (vol. ii., p. 21.)

The commencement of an attack of paralysis is generally marked by the occurrence of lassitude, sense of weight or of cold, numbness accompanied by weakness, a kind of weariness or soreness (brisure), unwonted stupidity, and some impediment to motion in the parts threatened with the disease, these symptoms often disappearing when the man gets heated at his work. (vol. ii., p. 26.) The author has never seen pain of the head or in the course of the vertebral column precede the paralysis, though he has sometimes observed more or less tremor, attended with some degree of stupor and heaviness before the attack. When this actually occurs, the loss of motion in the affected muscle or muscles is the first morbid phenomenon which appears, and usually from this period no effort of the will can excite even the most feeble contraction. In some rare cases the paralyzed muscle may yet be able to execute some obscure and uncertain motions, but M. Tanquerel contends that, notwithstanding that many authors both ancient and modern have advanced statements precisely the contrary to this, the instances in which even an imperfect power of motion and contraction seems to remain are more apparent than real, the movements which take place being performed by the assistance of other muscles not paralyzed. (vol. ii., p. 28.) This is a point which can only be determined by a close and accurate analysis of the motions in each individual case. Paralysis of some part of the superior extremities, especially of the wrists and fingers, was observed in ninety-seven cases, of the lower extremities in fifteen, of the intercostal muscles in two, and of the thoracic muscles generally in one case. The vocal organs were affected in thirty-one instances, of which fifteen were stammering or hesitation, and sixteen aphonia. With the exception of some cases in which the paralysis was general the extensor muscles only were affected, the reason of which, as M. Tanquerel remarks, does not appear. In five cases anæsthesia was observed at the same time in the parts deprived of motion, and in eight cases arthralgia. Although the loss of power is almost universally complete in the affected muscle, it does not arrive at that state until after a few days, but commences with simple numbness and slight tremor. The tremor assumes in no case the extent of alternate, almost spasmodic, contraction and resolution observed in mercurial tremors, but consists rather in a slight agitation of the muscles, and must be considered as the first degree of paralysis. (vol. ii., p. 32.) When the paralysis has been of long duration there is a flaccidity, a withering and extraordinary emaciation of the parts affected; the skin becomes blanched and sallow, sometimes livid, harsh, dry, loose, and flaccid; the cuticle scales off; the subcutaneous cellular tissue disappears, and the adipose tissue is entirely absorbed; the flesh feels flabby; the muscles become much decreased in size, and when the partial emaciation or atrophy has arrived at its ultimate stage the skin seems adherent to the bones, every prominence of which may be readily distinguished through the wasted muscles. The pulse, contrary to the opinion of M. Merat and others, who describe it as constantly vibrating, slow, and very strong, is correctly stated by the author to be weak, soft, easily compressible, and very slow. Neither in the head nor in the spinal column is there pain or other symptom to indicate any anatomical lesion of the nervous centres as the cause of the paralysis.

M. Tanquerel describes several varieties of lead paralysis, according as it affects different parts of the body, or different muscles or sets of muscles; most of these present nothing peculiar beyond what the local anatomical relations of the parts at once indicate. When the intercostal muscles, however, become the seat of the paralysis, a very rare occurrence, and seen only twice by the author who, moreover, has not been able to find any similar instances recorded, the symptoms are in the highest degree alarming. All at once, without previous physical lesion of the internal organs of the chest, the costal respiration is executed with the greatest difficulty, the ribs appearing almost entirely motionless. When the patient is required to make a great effort at inspiration the clavicles are evidently elevated, the rest of the thoracic parietes follow the movement, but the ribs are neither raised by themselves nor separated wider apart. The walls of the chest are considerably depressed; the action of the diaphragm, on the contrary, is greatly augmented, and in its alternate contractions it elevates the abdomen in an extraordinary manner. The respiration becomes noisy and the expectoration difficult; serous effusion accumulates in the bronchi, the lungs become congested, and death supervenes by asphyxia, in a manner analogous to what takes place in animals in which the pneumo-gastric nerves have been divided. During the whole of this scene the pulse is irregular, extremely rapid and small, the skin cool, the countenance anxious, the eyes staring, and the nostrils distended. The mind continues clear, the speech is short and hurried, and the voice whispering. (vol. ii., pp. 61-2.)

The progress of lead paralysis is in general slow and gradual; its duration, except in the case of its affecting the intercostal muscles when it is necessarily rapidly fatal, indefinitely prolonged. Usually the diagnosis is not difficult, being readily deducible from the history of the case, the exposure of the patient to the influence of lead, its complications with colic, from the local seat of the disease in certain muscles only, and more especially from its being confined to the extensors. There are cases, however, where it affects the whole of a limb and where its origin is obscure, in which it differs in no respect from ordinary paralysis produced by other causes; two or three such instances are mentioned by the author.

The prognosis is more or less unfavorable in relation to the extent and duration of the paralysis, to the importance of the parts affected, to the debility of the patient, and to the number of attacks previously experienced; where the paralysis is confined to some few of the muscles of a limb and appropriate remedies are timely applied, it is favorable; on the contrary, should the intercostal muscles be affected a fatal termination is the inevitable result. Little satisfactory information has been elicited as to the state of the nervous centres in lead paralysis, the principal morbid change observed in the inspections which have been performed being serous effusion beneath the membrane, considered by M. Tanquerel, and probably with justice, to have been poured out at the time of death. The local alterations noticed are similar to what is observed in the ordinary forms of paralysis, and result from the defective vitality in the parts affected. Thus the muscles are wasted and pallid or otherwise discoloured, scarcely preserving their original form, and resembling rather cellular tissue than muscular fibre; they become either so softened as to tear with the greatest facility, or else dry, mummified as it were, and almost of the nature of ligament. The caliber of the blood-vessels is diminished and they contain less blood than usual, while the nerves according to some authorities become atrophied. The following is the result of some microscopic examinations mentioned by the author. "1st. The spinal marrow, although more softened than the brain, exhibited the tubes discovered by Ehrenberg in a state of perfect integrity. 2d. The nerves distributed to the paralyzed muscles showed under the microscope their ordinary structure. 3d. The paralyzed muscles deprived of colour, exhibited under the microscope their proper fascicular structure with transverse striæ as usual, only colourless." (vol. ii., p. 80.)

Various methods of treatment have been proposed; but of these three only require notice, the treatment by sulphureted baths, by electricity, and by strychnine. In the first of these from five to six ounces of sulphuret of potash are required for each bath, which should be used only tepid, the patient remaining in it from three quarters of an hour to an hour. During this time he experiences nothing beyond a sensation of general warmth; on coming out of the bath, however, the limbs seem lighter, more supple, and easier to move; sometimes he is attacked with numbness, faintness, and severe headach; a general redness of the surface is observed and especially of the affected parts, which are often covered with a more or less abundant black matter, arising from the decomposition of the sulphuret of potash of the bath by the lead ingrained into the skin. In from a quarter to half an hour the benumbed limbs become heavy and unwieldy, but at the termination of three or four hours it is perceived that the movements become more regular, acquiring both force and steadiness. The author has seen only five cases of paralysis cured by this mode of treatment, used by itself, but he considers it a valuable adjunct to the other methods by electricity and the preparations of nux vomica, and recommends its employment as soon as these agents begin to produce their peculiar effects. (vol. ii., pp. 88-9.) The treatment of paralysis by electricity employed in various ways is well known in this country, and need not occupy us here. Fifteen of M. Tanquerel's cases affected with partial paralysis of the upper extremities were treated by electro-puncture; eight of these were completely cured in periods of time varying from one month to three months and eight days, in the other seven the treatment could not be persisted in from the supervening of inflammatory symptoms, or from the patient refusing to submit to it as long as was necessary to complete the cure. Strychnine was administered to forty patients, the whole of whom were either completely cured or their symptoms greatly alleviated, two months being the mean duration of the treatment. With the use of these remedial measures should be conjoined an appropriate regimen; dry warm apartments exposed to the sun, woollen clothing, nourishing food easy of digestion, with the moderate use of wine, beer, coffee, &c. exercise of the paralyzed parts, external frictions, &c.

IV. Anæsthesia from Lead. Twenty-three cases of anæsthesia were noticed by M. Tanquerel, in four of which the disease was deep-seated, in seven the loss of sensation was confined to the skin, and in twelve the eye was affected. In the eleven cases of deep-seated and superficial anæsthesia, three times there was paralysis of the corresponding muscles, four times the paralysis of motion and of sensation occupied different parts, and four times the loss of sensation was unaccompanied by loss of motion; in one case only did amaurosis and anæsthesia of the skin exist together. (vol. ii., pp. 201-2.) Of the cases of deep-seated anæsthesia there was loss of sensibility of the deltoid region in one; in another there was paralysis of the thigh with anæsthesia of the leg; in a third the power of motion was lost over the whole extent of the upper extremity, —the anæsthesia commenced at the fingers and extended to two thirds of the arm; in the fourth there was paralysis of the intercostal muscles and of the vocal organs, and at the same time loss of sensation of the neck and of the parietes of the thorax as far as the ensiform cartilage. In one of the cases of superficial anæsthesia there was paralysis of motion of the extensors of the wrist and fingers, the sensation of the back of the hand and fingers being preserved, while the palmar surface of the hand had entirely lost its sensibility, the flexor muscles of the wrist and fingers at the same time retaining their powers of motion. In another instance there was superficial anæsthesia with exalted sensibility of the parts beneath and paralysis of the corresponding muscles.

The lesion of sensation is always partial or of limited extent, sometimes confined to certain parts of the abdomen, of the chest or neck, sometimes occupying the limbs; it may be complete or varying in degree, frequently shifting its place, or varying in extent; when deep-seated it is less mobile than when confined to the skin. Usually it makes its attack suddenly and rapidly attains to its fullest extent, though occasionally it is preceded by a slight numbness. The treatment recommended is the sulphureted bath, and should this fail, frictions, epispastics, and irritants of various kinds, sudorifics, blisters, the cautery, or moxas applied either to the surface or along the vertebral column. Should these measures not succeed, which however is very rarely the case, recourse must be had to electro-puncture and strychnine, the exhibition of cathartics internally accompanying these measures throughout.

Amaurosis has been observed to occur as a consequence of lead poisoning by MM. Andral, Rognetta, Grisolle, Trousseau, and others; M. Tanquerel has had occasion to observe it twelve times. In nineteen cases which he has collected, five times the amaurosis was primary, that is, not preceded by any other form of lead poisoning, and fourteen times consecutive either to colic, arthralgia, or cerebral affection. Usually its attack is sudden and without premonitory symptoms; but in four instances it was preceded by frontal headach, and in another there was a gradual increase in the weakness of sight previous to complete amaurosis taking place. The amaurosis may be complete or imperfect only: in the former case there is considerable dilatation of the pupil and entire insensibility of the iris to the light, the membranes and humours retaining their transparency; in the latter case, which however is of less frequent occurrence, the power of distinguishing light from darkness is retained, and the patient believes that he sees objects through a dense mist. The pupil is moderately dilated and is yet somewhat sensible to light, the iris in fact is still somewhat contractile. The amaurosis is not always equal in degree in both eyes, and in this case it is not uncommon that slight strabismus is observed. The progress is in general rapid, the duration of the affection lasting from a few hours to a few days; once only is it said to have continued for years; the mean duration seems to be from four to six days. No anatomical lesion has been discovered in individuals dying while affected with amaurosis from lead, neither in the retina, in the optic nerve, nor in the brain. The treatment recommended consists in the repeated application of blisters to the neck, temples, behind the ears, and over the eyebrows; tartar-emetic ointment, setons, the cautery, and moxas, and in the event of these measures failing and the disease becoming of longer duration, the endermic application of strychnine to the temples, forehead, and superciliary region. Immediate and permanent cessation from employment in leadworks of any description is of course indispensable where so important an organ is concerned.

V. Cerebral Affections from Lead. The term *Encéphalopathie* is made use of by M. Tanquerel to designate those symptoms arising from the noxious influence of lead upon the animal economy, which would seem to depend more immediately upon some lesion of the brain. It includes, therefore, the various forms of cerebral affection, delirium, convulsions, epilepsy, coma, &c. which are observed in persons suffering from lead poisoning. Many of these affections have been long recognized as the result of the absorption of lead, though some authors, as M. Merat, have considered them in the light of complications rather than as actual symptoms of certain peculiar forms of lead disease. M. Tanquerel has met with seventy-two cases of these cerebral affections which he attributes entirely to the action of lead as the efficient cause.

He seems to think, however, that it requires the absorption of a larger proportion of lead particles to induce cerebral affection than is requisite for the production of colic, arthralgia, or paralysis; since he has only observed symptoms of this description in workmen exposed to the emanations from lead in large quantities. Those preparations of lead which are the most readily diffused, for instance, ceruse and red lead, are stated to give rise to cerebral affections the most frequently. It is necessary also that a certain time should elapse from the first exposure before the disease manifests itself. Of the seventy-two cases, ten occurred in persons who had been at work from eight to thirty days, thirty-four in workmen employed from one to nine months; the subjects of the remaining twenty-eight cases had been exposed to the emanations for various periods from one year to ten, twenty, and in one case fifty-two years, before becoming affected with cerebral symptoms. Six of the seventy-two persons attacked with encephalopathia had not previously suffered from any other form of lead disease; and ten others presented no trace of colic, arthralgia, or paralysis, at the time when they became affected with cerebral symptoms. Laennec, Dance, Andral, and others have also recorded instances of primary encephalopathia. In fifty-six cases which were accompanied with colic, the colic was slight or moderate in twenty-nine, and severe in twenty-seven. From these and other facts the author draws this inference, "that encephalopathia is one of the distinct forms of lead poisoning, and independent of colic and other diseases resulting from lead." (vol. ii., p. 273.)

It appears that in about a third of the cases certain premonitory symptoms may be observed to precede the more decided manifestation of cerebral affection, the most common of which is a peculiar, astonished, dull, or pensive expression. Pain of head varying in severity and extent, often accompanied with giddiness, watchfulness, or disturbed and broken sleep, noise in the ears, various affections of the vision, indefinable uneasiness, embarrassment and slowness of the mental faculties, stupor, &c. may one or all of them precede for some hours the attack of delirium, convulsion, or coma. Forty-two of M. Tanquerel's cases were preceded by colic, but this can scarcely be reckoned as a precursory symptom; it should however be observed that when colic which had previously been severe suddenly subsides, and any of the premonitory symptoms above mentioned appear, an attack of encephalopathia is to be dreaded.

M. Tanquerel establishes four distinct forms of the cerebral affection: 1st, that of delirium; 2d, the comatose; 3d, the convulsive; and 4th, where the delirium, coma, and convulsions are united. In the first of these, delirium forms the prominent and leading feature of the disease, and either assumes very much of the characters of the peculiar restless, talkative hallucination of delirium tremens, or the more violent symptoms attendant upon acute meningitis. The former of these states, constitutes the author's first variety, Quiet Delirium (*Délire tranquille*). Sometimes the patient remains quiet, with the features immoveable and the eyes fixed, with somewhat of an astonished air; sometimes the countenance is composed and the expression thoughtful; at other times again the eyes are turned upwards, the mouth is open, and the whole expression that of ecstacy. If spoken to, the answers are at first sensible, but after a time the ideas rapidly succeed each other, without apparent connexion. The patients are generally gay or sorrowful, loquacious or silent by turns; they toss their arms, throw off the clothes, endeavour to get out of bed, and are in a constant state of restlessness; hallucinations of sight and hearing, generally of a disagreeable character, harass them, and some few are affected with slight tremor, chiefly occupying the arms and face. (vol. ii. pp. 285-7.) In the latter, or second variety of M. Tanquerel, Furious Delirium (*Délire furieux*), the eyes are widely open, menacing, furious, or haggard; the features are contracted, the patient cries or shouts, swears, threatens, tears his garments to pieces, bursts through the restraints by which he was confined, rushes through the wards, and in short is in a state of complete and raging insanity. The delirium, in many of these cases, is accompanied by spasmodic contractions of the muscles of the face, distortion of the eyes, closing and chattering (*craquement*) of the jaws, catchings of the tendons, or tremors of the limbs. Notwithstanding all this disturbance of the nervous system, it is yet possible occasionally to obtain rational replies to questions which do not require much exertion to comprehend their import. Once only did M. Tanquerel witness the patient carry his hand frequently to the forehead and complain of pain, when interrogated upon this point. (pp. 288-90.) The progress of the delirium, whether tranquil or furious, is of inconceivable irregularity, varying from instant to instant without order; sometimes, however, coming on in paroxysms. In both varieties there is frequent alternation, with a kind of muttering somnolence, usually of no long duration, though in some rare cases the interval between the paroxysms of loquacious, or of furious delirium lasts for several hours, which is always a favorable sign. When the delirium is about to terminate favorably, a long deep sleep, which must not be confounded with the state of somnolency, supervenes, at the close of which a complete and often unlooked for amelioration is observed.

The following is what M. Tanquerel considers to be the most regular and complete progress of the delirious form of encephalopathia:

"The cerebral disease commences by the tranquil delirium, at the end of a certain time paroxysms of fury take place; at a later period somnolency occurs, after which it is only the tranquil delirium which comes on at intervals, more or less approximate. At length genuine sleep succeeds the somnolency, at the close of which the patient becomes almost perfectly rational. From this time he is strongly disposed to sleep, which he is unable to resist. Exhausted and with his limbs as if bruised, he still preserves a somewhat astonished expression of countenance." (vol. ii. p. 294.)

Of the seventy-two cases of encephalopathia, eighteen belonged to this form of the affection.

The second, or comatose form, characterized by coma, more or less profound, occurred in six instances. Two varieties of this form are also distinguished by M. Tanquerel, according as the coma exists simply in a greater or less degree, or as it is complicated with a low delirium appearing in the course of the disease. In the latter case there is a partial rousing from the comatose state, during which the eyes are opened, and a few unintelligible words repeatedly muttered, and some degree of restlessness manifested. These two varieties of comatose encephalopathia may occur either alternately or separately during the course of the disease, and, as it seems, observe little or no regularity in their appearance or disappearance. "It is this form of encephalopathia," says M. Tanquerel, "which the least frequently presents precursory symptoms, or those which, when they do occur, offer nothing peculiar; its hasty and instantaneous development greatly serves to establish the diagnosis." Subsequently, however, he states that "the coma usually appears only consecutively to one or more attacks of epilepsy, and more rarely at the close of violent paroxysms of furious delirium." (v. ii. p. 297.)

Convulsions are of very frequent occurrence in the cerebral affections resulting from lead poisoning, though rarely observed isolated. Five varieties of the convulsive form are described: 1st. Partial convulsions, in which the face, or one side, or one or more of the limbs are agitated by a series of quick shocks, or there may be spasm of longer or shorter duration. 2d. General convulsions, in which the shocks extend to the whole body, affecting the face and superior extremities most severely, and distinguished from epilepsy by the consciousness being partially retained; several of these convulsive attacks were preceded by excessive pains of the limbs, and very violent colics. 3d. Epilepsy, the most common form in which cerebral disturbance, from the deleterious agency of lead, evinces itself. Thirty-six cases of epilepsy from lead were observed, the paroxysms lasting from two to thirty minutes each. In no one of these cases was there any trace of *aura epileptica* detected, and in one only did the patient utter a cry at the commencement of the attack. At the close of each paroxysm there followed either a comatose state, more or less profound, or one or other of the forms of delirium before noticed, which usually lasted until a fresh paroxysm of epilepsy came on, or the paroxysms of epilepsy succeeded each other, almost without interval. 4th. Epileptic convulsions, a term applied by the author to designate loss of sensation, and clonic spasms in the limbs and trunk, analogous to the convulsed state of epilepsy but differing in degree, and usually accompanied neither by foaming at the mouth nor by stertorous respiration. These spasms are almost continuous, and may last from one to twenty-four hours. 5th. Catalepsy, which has been observed only twice by the author; the appearance of a patient thus affected, is that of a person in a tranquil sleep; no sign of sensation is evinced, even on burning the skin, and it is impossible to arouse or fix his attention. The fingers, hands, forearms, arms, legs, and thighs, placed without support, in no matter what position, whether uneasy or otherwise, remain fixed in that situation for some seconds, or even one or two minutes; then they oscillate a little, and at length fall upon the bed. This state alternates with one in which there is some restlessness of motion and return of sensation, the superior extremities then resisting the attempt to place them in any fixed posture, or to move them from that in which they are found.

In the fourth form of encephalopathia there is a combination of all the symptoms occurring in the three other forms; it presents therefore the type of the disease. The utmost irregularity would seem to prevail in the severity and mode of succession of the different states of delirium, coma, and convulsive paroxysms. M. Tanquerel gives the following as the most usual and most regular.

"The patient is first seized with delirium, sometimes so slight as to escape the cognizance of the physician; at the end of some hours, and even sometimes after one or two days, an attack of epilepsy supervenes, at the close of which the patient falls into a drowsy state for some minutes, then seems to arouse to talk at random throughout the day; the delirium, either tranquil or furious, is then more marked than before the convulsive attack. The same day, the following night, or on the morrow, one or more attacks of epilepsy again supervene. After each paroxysm the drowsiness is deeper and more prolonged. It is interrupted occasionally only by a partial rousing of some minutes, during which the patient mutters a few words, to resign himself anew to sleep. If the paroxysms of epilepsy are frequently renewed, the coma becomes very deep, and death follows. In the contrary case, the patient seems all at once to arouse from his drowsiness, at the end of some hours or of a day." (vol. ii. p. 311.)

With these symptoms, more especially denoting the existence of cerebral disease, there are others combined which are found more or less in every form of the cerebral affection. There is usually some complication with one or more of the other forms of lead poisoning, existing either immediately before or during the attack of encephalopathia. Once in fourteen times paralysis was observed immediately to precede the cerebral symptoms, and once in seven times it appeared during their course. Amaurosis is a pretty frequent concomitant of encephalopathia, and has been observed by M. Tanquerel in ten cases. The circulation frequently remains unaffected in the midst of the severe disturbance of the nervous system, and the pulse is in many cases full, regular, and numbers not more than from seventy to eighty. Usually, however, the pulse is accelerated as the disease advances, often becoming extremely rapid, and considerably depressed; during the coma it sometimes becomes slow and hard. In one case only was there any inflammatory appearances observed on the blood drawn from a vein; in twenty others it presented no appreciable alteration. The tongue was commonly natural, but became dry after long-continued furious delirium.

The duration of the disease varied according to the type which it assumed; the delirium usually disappeared at the end of the third or fourth day, but lasted in different cases from a few hours to seventeen days. The epilepsy recurred in paroxysms at intervals of greater or less duration, from some minutes to six or seven days, and the number of paroxysms occurring in the space of twenty-four hours varied from one to thirty-four. The comatose form usually lasted from one to three days; while the duration of the fourth form of the cerebral disease varied between four days and seventeen days. Of the seventy-two cases reported by M. Tanquerel, sixteen proved fatal, and fifty-six were cured, whilst of eighty-nine cases which he has collected from other authors, sixty-one terminated in death. In no case observed by M. Tanquerel, did the disease run into meningitis or encephalitis, nor has he been able to find any authentic case of such a termination recorded elsewhere. The subjoined table shows the mortality of the different forms of the disease as observed by the author and other writers, (vol. ii. p. 345.)

| | | Tanquerel. | | Other authors. | |
|---|---|---|---|---|---|
| | | Cures. | Deaths. | Cures. | Deaths. |
| 1st form— | Delirium - - | 16 | 2 | 14 | 15 |
| 2d | Coma - - | 3 | 3 | 2 | 5 |
| 3d | Convulsion - | 12 | 2 | 7 | 25 |
| 4th | Complication - | 25 | 9 | 5 | 16 |
| | | — | — | — | — |
| | | 56 | 16 | 28 | 61 |

No inference, however, can be drawn from the cases of writers in general as to the relative mortality of the whole or of any individual form, since many of the milder cases have probably not been recorded; with respect to those of M. Tanquerel it may be observed that although the total number is small, there can be no doubt of the correctness of the conclusion drawn from them, that the comatose and complicated forms are the most to be dreaded. Deep coma, that is, the first variety of this form described by the author, is almost always fatal. In the other three forms of encephalopathia, the most unfavorable prognosis is indicated by the general severity of the symptoms, furious delirium marking the danger in the first, and the occurrence of epilepsy in the third, while in the fourth the predominance of delirium over coma or epilepsy in the general features of the case becomes a favorable sign, especially if it continues for three or four days.

A careful diagnosis is drawn up of the several forms of encephalopathia from cerebral affections in general and from other diseases in which the brain and nervous system may become more or less implicated. Those which are most liable to be confounded with cerebral disease from lead poisoning are nervous delirium, delirium tremens, coma resulting from cerebral disturbance, mental alienation, and ordinary epilepsy. (vol. ii., p. 343.) The similarity of the mild variety of delirium to delirium tremens we have already had occasion to point out, that of the paroxysms of the furious delirium to some states of mental alienation is no less striking. In fact, the disease is mania, only induced by a particular cause. In most instances the history of the case and the occupation of the patient will materially tend to establish the diagnosis. The greatest difficulty will be found with delirium tremens occurring in workmen exposed to the emanations from lead, many of whom are hard drinkers; in such cases, the best mode of drawing the requisite distinction is by a close and careful search after other symptoms of lead poisoning, any tendency to coma especially pointing out the true origin of the affection to be from this last cause.

From a careful comparison of the anatomical appearances in sixteen inspections of persons dying from encephalopathia performed by himself with those recorded by several others, M. Tanquerel establishes, first, that in twenty-one cases there existed a flattening, a crowding together (tassement) of the cerebral convolutions, with increased or diminished cohesion of the cerebral pulp, with increase or diminution in the volume of the cerebral mass; secondly, that in nineteen cases yellow discoloration of the cerebral matter was found; and thirdly, that in thirty-two cases there was observed, and only occasionally, slight serous infiltration, sanguineous injection of the membranes, a diminution of consistence, especially in the medullary matter, without change of colour, or, finally, paleness (*décoloration*) or loss of colour in the cerebral substance. (vol. ii., pp. 356-7.) Some of these appearances are considered by the author to be altogether foreign to the disease, others as the effects of the last struggles of the patient, others as secondary phenomena connected with the lead poisoning but exercising no influence in the causation of the symptoms. We regret that we are precluded from analyzing the cases at length, since, as it would seem to us, the hypertrophied state of the brain so frequently observed, though by no means universally present, has much to do with the production of a part at least of the symptoms observed during life. The dirty earthy yellow discoloration of the substance of the brain, it is admitted, (vol. ii., p. 360,) cannot be considered to be otherwise than allied to the peculiar jaundiced state which forms one of the characteristics of primary lead poisoning, mentioned among the premonitory symptoms. MM. Devergie and Guibourt have detected lead in an appreciable quantity in the brains of two subjects dying of encephalopathia, which were submitted to analysis, and in a microscopical investigation by M. Gluck of a portion of the brain analyzed by M. Guibourt, the white matter of the brain was found remarkably altered in its minute structure. "In many parts of the white matter of the brain," observes M. Gluck, "not only at the surface but also in the interior, the tubes discovered by Ehrenberg appear as if shrunk up; in other points these tubes are well preserved." He subsequently remarks that the alteration could not be attributed to putrefaction, for that the spinal marrow, though much more softened, had notwithstanding preserved its structure. (vol. ii., pp. 362-3.)

Could any inference be drawn from the results of the cases recorded by M. Tanquerel and those which he has collected from other sources, it would appear that the treatment followed in the former was by far the more efficacious; but, as we have before remarked, the milder cases are probably not included among the selected instances in sufficient proportion. In thirty-four of M. Tanquerel's cases, eight of which belonged to the delirious form, two to the comatose, and twenty-four to the fourth form or that in which delirium, coma, and convulsion were all present, no active treatment was had recourse to; one only proved fatal. Of six cases of the delirious form treated by opium, on account of the analogy which exists between this form and delirium tremens, four were fatal and the other two more prolonged in duration than the cases left to the efforts of nature; we must remark, however, that in these cases the opium was by no means efficiently administered. In four cases in which bleeding was had recourse to, the symptoms were manifestly aggravated by its employment, and of four instances where blisters to the scalp, ice and other remedies termed revulsive were used, two terminated fatally. Four patients affected with the convulsive form of the disease were treated by means of opium without benefit, and two of these died. In twenty-one the purgative and other methods prescribed against colic were followed; six of these died, and the disease was protracted in the others. Cold affusion was employed three times: one of the patients died, the two others did not recover until several days after the discontinuance of the remedy. From these data it would seem that in accordance with the opinion of M. Rayer, all active treatment should be avoided, as tending only to increase the cerebral disturbance already existing.

We have hitherto given a close analysis of the contents of these volumes; the concluding portion, in which are some excellent directions to be followed in the different circumstances of exposure to the action of lead, we are compelled to pass over. This, however, we the less regret, as many of the precautions recommended are in actual operation in this country, and others are ably enforced in works to which every one has access.

